# Corneal Biomechanical Parameters and Central Corneal Thickness in Glaucoma Patients, Glaucoma Suspects, and a Healthy Population

**DOI:** 10.3390/jcm10122637

**Published:** 2021-06-15

**Authors:** Mª. Ángeles del Buey-Sayas, Elena Lanchares-Sancho, Pilar Campins-Falcó, María Dolores Pinazo-Durán, Cristina Peris-Martínez

**Affiliations:** 1 Department of Ophthalmology, Hospital Clínico Universitario “Lozano Blesa”, Avda. San Juan Bosco 15, 50009 Zaragoza, Spain; madelbuey@gmail.com; 2 Aragón Health Research Institute (IIS Aragon), Avda. San Juan Bosco 13, 50009 Zaragoza, Spain; 3 Aragon Institute of Engineering Research (13A), University of Zaragoza, Mariano Esquillor s/n, 50018 Zaragoza, Spain; elanchar@unizar.es; 4 Centro de Investigación Biomédica en Red en Bioingeniería, Biomateriales y Nanomedicina (CIBER-BBN), 28029 Madrid, Spain; 5 Department of Analytical Chemistry, Faculty of Chemistry, University of Valencia, 46100 Valencia, Spain; pilar.campins@uv.es; 6 Ophthalmic Research Unit “Santiago Grisolía”, Foundation for the Promotion of Health and Biomedical Research of Valencia FISABIO, 46017 Valencia, Spain; pinazoduran@yahoo.es; 7 Ophthalmology Department, University of Valencia, 46019 Valencia, Spain; 8 FISABIO Oftalmología Médica (FOM), Foundation for the Promotion of Health and Biomedical Research of Valencia (FISABIO), 46015 Valencia, Spain; 9 Aviñó Peris Eye Clinic, 46001 Valencia, Spain

**Keywords:** glaucoma, corneal hysteresis, ocular inflammation, corneal biomechanics, ocular biomarkers

## Abstract

Purpose: To evaluate and compare corneal hysteresis (CH), corneal resistance factor (CRF), and central corneal thickness (CCT), measurements were taken between a healthy population (controls), patients diagnosed with glaucoma (DG), and glaucoma suspect patients due to ocular hypertension (OHT), family history of glaucoma (FHG), or glaucoma-like optic discs (GLD). Additionally, Goldmann-correlated intraocular pressure (IOPg) and corneal-compensated IOP (IOPcc) were compared between the different groups of patients. Methods: In this prospective analytical-observational study, a total of 1065 patients (one eye of each) were recruited to undergo Ocular Response Analyzer (ORA) testing, ultrasound pachymetry, and clinical examination. Corneal biomechanical parameters (CH, CRF), CCT, IOPg, and IOPcc were measured in the control group (*n* = 574) and the other groups: DG (*n* = 147), FHG (*n* = 78), GLD (*n* = 90), and OHT (*n* = 176). We performed a variance analysis (ANOVA) for all the dependent variables according to the different diagnostic categories with multiple comparisons to identify the differences between the diagnostic categories, deeming *p* < 0.05 as statistically significant. Results: The mean CH in the DG group (9.69 mmHg) was significantly lower compared to controls (10.75 mmHg; mean difference 1.05, *p* < 0.001), FHG (10.70 mmHg; mean difference 1.00, *p* < 0.05), GLD (10.63 mmHg; mean difference 0.93, *p* < 0.05) and OHT (10.54 mmHg; mean difference 0.84, *p* < 0.05). No glaucoma suspects (FHG, GLD, OHT groups) presented significant differences between themselves and the control group (*p* = 1.00). No statistically significant differences were found in the mean CRF between DG (11.18 mmHg) and the control group (10.75 mmHg; mean difference 0.42, *p* = 0.40). The FHG and OHT groups showed significantly higher mean CRF values (12.32 and 12.41 mmHg, respectively) than the DG group (11.18 mmHg), with mean differences of 1.13 (*p* < 0.05) and 1.22 (*p* < 0.001), respectively. No statistically significant differences were found in CCT in the analysis between DG (562 μ) and the other groups (control = 556 μ, FHG = 576 μ, GLD = 569 μ, OHT = 570 μ). The means of IOPg and IOPcc values were higher in the DG patient and suspect groups than in the control group, with statistically significant differences in all groups (*p* < 0.001). Conclusion: This study presents corneal biomechanical values (CH, CRF), CCT, IOPg, and IOPcc for diagnosed glaucoma patients, three suspected glaucoma groups, and a healthy population, using the ORA. Mean CH values were markedly lower in the DG group (diagnosed with glaucoma damage) compared to the other groups. No significant difference was found in CCT between the DG and control groups. Unexpectedly, CRF showed higher values in all groups than in the control group, but the difference was only statistically significant in the suspect groups (FHG, GLD, and OHT), not in the DG group.

## 1. Introduction

Corneal biomechanics studies the balance and deformation of the corneal tissue subjected to any external action. This scientific discipline explores the function and the inner structure of the cornea and endeavors to establish some physical-mathematical bases that define it [1]. The possible practical applications range from the diagnosis and assessment of certain pathologies [2,3,4,5] to the prediction of response to corneal surgical procedures [6,7,8,9].

The Ocular Response Analyzer, ORA (Reichert), was the first device to measure the biomechanical properties of the cornea in vivo. It can determine some parameters of the structure and viscoelastic properties of the cornea, and also intraocular pressure (IOP) [10]. Corneal biomechanical parameters measured by ORA are corneal hysteresis (CH) and corneal resistance factor (CRF), as well as noncontact intraocular pressures, such as the Goldmann-correlated intraocular pressure (IOPg) and corneal-compensated intraocular pressure (IOPcc). Several studies have tried to set biomechanical parameters to help improve prediction of corneal ectasia, refractive surgery pre- and post-operative evaluation, corneal pathologies, and glaucoma. It has been shown that CH is lower after refractive surgery in corneal ectasia, such as in keratoconus, Fuchs’ dystrophy, and glaucoma patients [11,12,13,14,15]. Glaucoma is a chronic and progressive optic neuropathy characterized by loss of the retinal nerve fiber layer, progressive optic disc damage, and the development of characteristically evolving visual field defects. It is associated, although not in all cases, with an increase in IOP. The prevalence of glaucoma is 1.5%–2% in individuals over 40 years of age and even higher in those over 60 years of age. Glaucoma is an especially relevant ocular pathology, since it is the second cause of irreversible blindness after diabetic retinopathy. The most common type, accounting for 60% of glaucoma cases, is primary open-angle glaucoma (POAG). It is usually bilateral, although frequently asymmetric, and the chamber angle is open and is not related to another ocular disorder. The exact etiology of POAG is unknown, but there are some risk factors, including IOP, family history, increasing age, race, myopia, and cardiovascular status [15,16]. It is currently known that, although an increase in IOP is the most important risk factor for suffering from glaucoma and the only one on which we can act at the moment, it is not the only determining factor. Some facts support these claims, suggesting that other risk factors should be considered: There are patients who present an IOP of over 21 mmHg (even 30 mmHg) but do not present alterations in the optic nerve or in the visual field. They are called ocular hypertensive or glaucoma suspects, and although some develop glaucoma (40% in 10 years), others remain unaffected despite elevated intraocular pressure values. Another group of patients present visual field examination and optic nerve head alterations typical of glaucoma, with normal IOP values or even lower than usual, a circumstance that we call low-tension glaucoma or normal-tension glaucoma [17]. It has been reported that 45% of treated glaucoma patients had glaucomatous progression in visual fields despite an average 25% decrease in IOP [18]. Even so, IOP is the only factor on which we can act to stop the progress of the disease and to which all anti-glaucoma treatments have been directed so far.

In the last decade, the biomechanical properties of the cornea have received increasing attention, particularly corneal hysteresis (CH). Corneal biomechanics provides insight into the behavior of the cornea and could reflect the vulnerability of the optic nerve structures to glaucoma. Thus, CH could become a potential glaucoma biomarker, which could serve as a risk indicator of progression. Besides CH, a new compensated IOP (IOPcc) would show a more real IOP value. The analysis, study, and assessment of corneal biomechanical properties, IOPcc, and corneal thickness obtained with the ORA in normal eyes, in eyes with glaucoma, and in eyes with suspected glaucoma, provide new data that can help identify patients with a higher risk of glaucoma progression.

The purpose of this study was to evaluate and compare CH and central corneal thickness (CCT) measurements between a healthy population (controls), patients diagnosed with glaucoma (DG) in treatment for IOP control, and glaucoma suspect patients due to ocular hypertension (OHT) or other risk factors, such as family history of glaucoma (FHG) or glaucoma-like optic discs (GLD) [19]. Additionally, the corneal resistance factor (CRF), Goldmann-correlated intraocular pressure (IOPg), corneal-compensated IOP (IOPcc), and CCT are evaluated and compared between the different groups of patients. Furthermore, the study of a large control group defines the mean values of corneal biomechanical properties and IOP in the healthy Spanish population.

## 2. Methods

This is a prospective analytical-observational study that was performed at the Anterior Segment section of the Department of Ophthalmology, Hospital Clínico Universitario Lozano Blesa, Saragossa (Spain). The study was approved by the Institutional Review Board of the University of Saragossa Faculty of Medicine and was conducted in accordance with the Declaration of Helsinki for research involving human subjects. The studywas approved by the Ethics Committee for Clinical Research of Aragon (CEICA 18/2017) and the date of approval was 25 October 2017. Written informed consent was signed by all participants.

### 2.1. Subjects

Subjects included in this study were healthy persons without a diagnosed ocular pathology (controls), patients diagnosed with glaucoma (DG), and glaucoma suspect patients who were undergoing a study for early diagnosis due to ocular hypertension (OHT), family history of glaucoma (FHG), or glaucoma-like optic discs (GLD).

Subjects excluded from this study were patients with serious general diseases, such as recent surgery, malignant neoplastic pathology, collagen diseases, immunological diseases, metabolic stress due to moderate-severe renal failure, decompensated diabetes mellitus, altered nutritional status, or any general situation of the patient that could compromise the results of the evidence. Further exclusion criteria were patients with other ocular pathologies, such as severe dry eye syndrome, corneal chemical burn, infectious and inflammatory diseases of the cornea, conjunctiva, uvea and sclera; corneal ectasia or dystrophies; acute pathologies and postsurgical states, such as retinal detachment, acute glaucoma, proliferative diabetic retinopathy, iris rubeosis, need for valve implants due to glaucoma, patients undergoing any corneal surgery, or patients who presented signs of corneal pathology.

### 2.2. Examination Techniques

All participants underwent exhaustive ophthalmologic examination, including a review of medical history, slit-lamp biomicroscopy, best-corrected visual acuity, IOP measurement using Goldmann applanation tonometry (GAT), and retinal ophthalmoscopy examination. The clinical examinations necessary for the diagnosis and evolutionary assessment of the patients were carried out following an action protocol specific to the ocular pathology group to which they belonged. The entire battery of diagnostic devices typical of a hospital ophthalmology department were available to us to perform this study. These included a refractometer, indirect ophthalmoscope, optical coherence tomography (OCT), ORBSCAN ocular topography, endothelial analysis with a non-contact specular microscope, and standard automated perimetry (SAP).

In the group of healthy patients, topographic exploration with ORBSCAN was also performed on those patients with refractive defects (to rule out evident keratoconus or subclinical keratoconus), as was specular microscopy on patients with doubtful biomicroscopy or high corneal thickness suspected of edema. If any corneal pathology was evidenced, the patient was excluded from the study.

In the DG group, functional diagnostic tests with standard automated perimetry (SAP), the Swedish Interactive Threshold Algorithm (SITA), 24–2 testing of the HVS Analyzer II, model 750i (Carl Zeiss Meditec, Inc., Dublin, CA, USA), and structural tests (fiber and papilla OCT) were performed to assess glaucomatous damage.

Examination was completed with gonioscopy, stereoscopic optic disc photography, functional tests with SAP, and structural tests (OCT of fibers and papilla) in the glaucoma suspect groups (OHT, FHG, or GLD). If there was evidence of unknown glaucomatous damage, treatment was prescribed, and the patient was excluded from the study. Ocular hypertension was defined as IOP equal to or higher than 21 mmHg with a normal visual field and optic nerve. Family history of glaucoma was determined as at least one of the parents with diagnosed glaucoma. Glaucoma-like optic discs were defined as having a vertical cup-to-disc ratio greater than 0.5 or asymmetry of cupping between the two eyes in patients with normal IOP and visual fields and open angles.

All patients were explored with Reichert’s Ocular Response Analyzer (ORA), both to determine their biomechanical properties (CH, CRF) and to determine their Goldman equivalent IOP (IOPg) and compensated IOP (IOPcc). The central corneal thickness (CCT) was determined from the pachymetry performed with the ultrasonic pachymeter incorporated in the ORA. All ocular examinations, including ORA, were performed on the same day. Four consecutive ORA measurements were determined in both eyes. Then the results were averaged. One eye of each patient was chosen at random.

### 2.3. Statistical Analysis

A total of 1065 patients (one eye of each) were recruited in this prospective analytical-observational study. Normal subjects comprised the control group (*n* = 574), who were studied alongside the DG (*n* = 147), FHG (*n* =78), GLD (*n* = 90), and OHT (*n* = 176) diagnosis groups. Microsoft Excel was used to collate data including patient age and gender, diagnosis group, CH, CRF, IOPg, IOPcc, and CCT. The IBM SPSS (version 17) statistical package was used for data analysis. An analysis of variance (ANOVA) was carried out for all the dependent variables according to the different diagnostic categories, with multiple comparisons to distinguish between the diagnostic categories in which there were differences, taking *p* < 0.05 as statistically significant.

## 3. Results

### 3.1. Subjects

**Figure 1:**
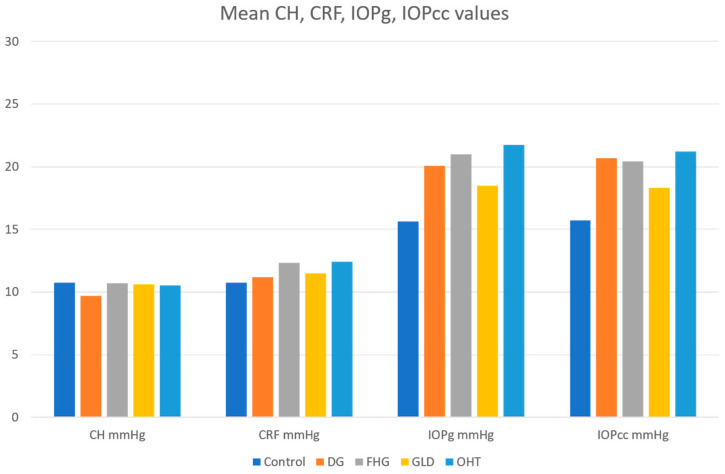
Plot of meanCorneal Hysteresis (CH), Corneal Resistance Factor (CRF), Goldmann-correlated intraocular pressure (IOPg) and corneal-compensated IOP (IOPcc) values in all groups. DG—diagnosed glaucoma; FHG—family history of glaucoma; GLD—glaucoma-like optic discs; OHT—ocular hypertension.

**Figure 2:**
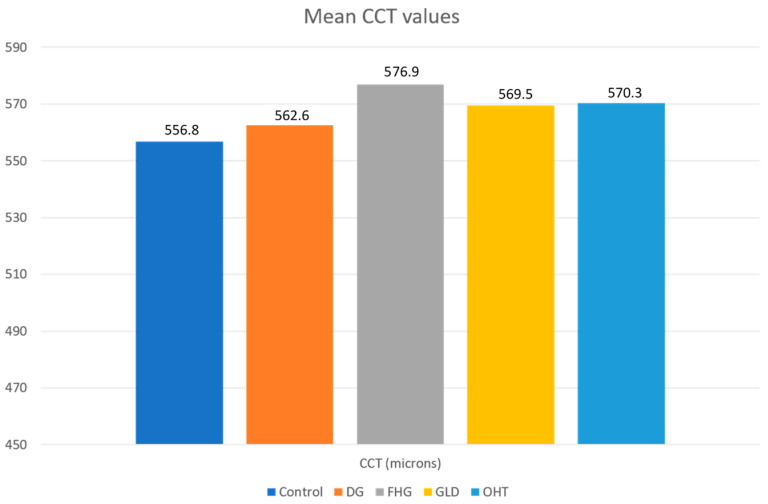
Plot of mean Central Corneal Thickness (CCT) values in all groups. DG—diagnosed glaucoma; FHG—family history of glaucoma; GLD—glaucoma-like optic discs; OHT—ocular hypertension.

The study included one eye of each of the 1065 patients. Table 1 summarizes the basic demographic information for the patients included in the five different diagnosis groups. A higher percentage of females was present in the FHG (71.8%) and GLD groups (78.9%) compared to the control (50.7%), OHT (54%), and DG (44.2%) groups. The control group patients were younger, on average, than the patients included in the other four groups. After carrying out a comparative study of the control group with the pathological groups, we found the following results for each variable using the ORA (CH, CRF, IOPg, IOPcc, and CCT), which are summarized in Table 2 and Figure 1 and Figure 2.

#### 3.1.1. CH

**Figure 3:**
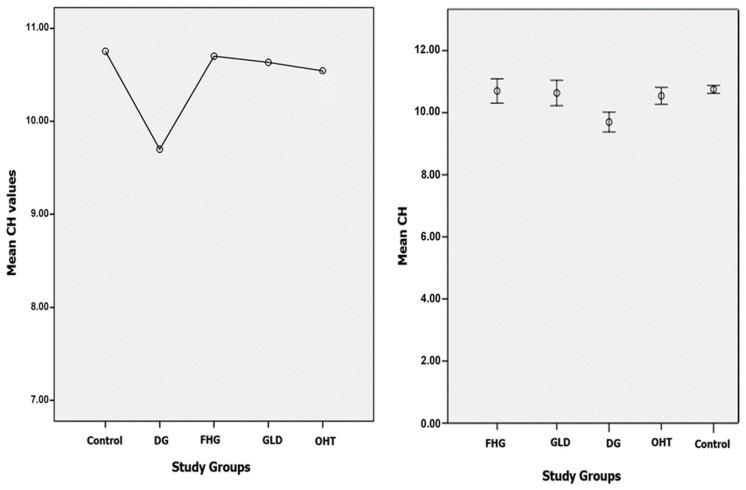
Comparison of mean values (**left**) and the scatter graphs (**right**) of the CH variable in all groups. DG—diagnosed glaucoma; FHG—family history of glaucoma; GLD—glaucoma-like optic discs; OHT—ocular hypertension.

The mean CH in DG patients (9.69 ± 1.9 mmHg) was lower than in the control group (10.75 ± 1.5 mmHg). Additionally, the mean CH in DG patients was lower than in all glaucoma suspect groups: FHG, GLD, and OHT (10.70 ± 1.7, 10.63 ± 1.9, and 10.54 ± 1.8 mmHg, respectively). The scatter graphs for the CH variable according to the groups studied show a minimal dispersion in the control group and a greater dispersion in the DG group and in the glaucoma suspect groups (FHG, GLG, and OHT). Figure 3 shows the comparison of the mean values (left) and the scatter graphs (right) of the CH variable in all groups. An inferential statistical study was carried out to verify the significance of these differences, using the ANOVA multiple comparisons test (Table 3), which shows the existence of statistically significant differences (*p* < 0.05) for the CH variable comparing the DG group with the rest of the groups. No significant differences were found between the control group and the glaucoma suspect groups (*p* = 1.00). The FHG, GLD, and OHT groups showed no significant differences between themselves (*p* = 1.00).

#### 3.1.2. CRF

The glaucoma suspect groups (FHG, GLD, and OHT) showed higher CRF values (12.32 ± 1.9, 11.50 ± 1.9, and 12.41 ± 1.8 mmHg, respectively) than the control group (10.75 ± 1.6 mmHg) with statistically significant differences (*p* < 0.001, *p* < 0.05, and *p* < 0.001, respectively). No statistically significant differences were found between the DG group and the control group. In the comparative study of the DG group versus the glaucoma suspect groups (FHG, GLD, and OHT), the FHG and OHT groups presented higher CRF values and the differences were significant (*p* < 0.05 and *p* < 0.001, respectively) (Table 4).

#### 3.1.3. IOPg

The DG group and the glaucoma suspect groups (FHG, GLD, and OHT) presented higher IOPg values (20.07 ± 3.6, 21.01 ± 4.0, 18.48 ± 3.6, and 21.76 ± 3.6 mmHg, respectively) than the control group (15.63 ± 3.1 mmHg) with statistically significant differences (*p* < 0.001). The DG group had significantly lower IOPg than the OHT group (*p* < 0.05) and significantly higher IOPg than the GLD group (*p* < 0.05). The GLD group presented significantly lower IOPg values than the DG (*p* < 0.05), FHG (*p* < 0.001), and OHT (*p* < 0.001) groups (Table 5).

#### 3.1.4. IOPcc

The DG group and the glaucoma suspect groups (FHG, GLD, and OHT) presented higher IOPcc values (20.68 ± 3.7, 20.41 ± 3.8, 18.30 ± 3.9, and 21.22 ± 3.8 mmHg, respectively) than the control group (15.72 ± 3.0) with statistically significant differences (*p* < 0.001). When comparing the DG group with the glaucoma suspect groups, the IOPcc values were found to be significantly lower in the GLD group (*p* < 0.001). The OHT group presented no differences when compared with the DG group, since the IOPcc values were lower than those of IOPg in this group (Table 6).

#### 3.1.5. CCT

The DG group and the glaucoma suspect groups (FHG, GLD, and OHT) presented higher CCT values (562.6 ± 39.6 μ, 576.3 ± 38.3 μ, 569.51 ± 31.5 μ, and 570.34 ± 34.7 μ, respectively) than the control group (556.8 ± 35.3 μ), but only the FHG and OHT groups exhibited statistically significant differences (*p* < 0.05). No significant differences were found in the comparative analysis of the DG group with respect to the FHG, GLD, and OHT groups (Table 7).

## 4. Discussion

### 4.1. Control Group

This work provides reference values for corneal biomechanical parameters (CH and CRF), IOPg, IOPcc, and CCT for the healthy Spanish population because it studied such a large control group (574 eyes). It should be noted that the existence of ocular pathologies (clinical or subclinical keratoconus, glaucoma, corneal dystrophies, and so on) in the control group was ruled out, and the study shows the results of a population with a wide age range. The mean age of the group of healthy control patients was 38.7 years, similar to that of other published studies [11,14,20,21]. The standard deviation of 15.54 in the age range (9–84 years) shows an age range of the control group subjects greater than that of other published works [10,22]. Mean values of CH (10.75 mmHg), CRF (10.75 mmHg), CCT (556.8 µ), IOPg (15.63 mmHg), and IOPcc (15.72 mmHg) hwere obtained. Means of the CH values obtained in other studies have ranged from 9.6 to 11.4 mmHg, but it should be taken into account that disparate population groups were studied regarding race and age, and moreover, some of them were carried out on a population of subjects with high refractive errors [23,24,25,26]. The mean CH obtained by Shah et al. [13] in healthy subjects was 10.7 ± 2.0, which is similar to that obtained in our work (10.7 ± 1.5). In another European study carried out on the eyes of healthy subjects [27], mean values of 10.7 ± 1.8 mmHg were obtained for the CRF, of 10.6 ± 1.6 mmHg for CH, of 15.9 ± 3.9 mmHg for IOPg, and of 16.2 ± 3.7 mmHg for IOPcc. It can be seen that they are similar to those obtained in our work, although in their study, they did not rule out the existence of possible subclinical keratoconus.

The ORA can determine IOPg values correlated to GAT, as well as IOPcc values, and it is less influenced by corneal biomechanical properties. In our study, we found that the control group of healthy patients, with normal biomechanical properties, presented similar mean IOPg and IOPcc values (15.6 mmHg and 15.7 mmHg, respectively). These values are similar to the GAT means reported in the literature for the healthy population, which are around 15.5 mmHg. We believe that these results, obtained from a large control group, validate the ORA as a tonometer, since the value means were determined in a large group of a healthy population, in which tests, such as topography and pachymetry, were performed that ruled out corneal subclinical alterations. Ping-Bo et al. [28] conducted a study using the ORA on 296 eyes of 158 healthy patients, classified into three groups according to their CCT (<520 µ, 520–580 µ, >580 µ), and found the following mean IOP values (IOPg: 14.95 ± 2.99 mmHg, IOPcc: 15.21 ± 2.77 mmHg, and GAT: 15.22 ± 2.77 mmHg), which are similar to those obtained in the control group of our work. When analyzing the values according to the CCT, they concluded that IOPcc measurements with ORA were only affected to a small extent by CCT, and they are probably much closer to the true value of the IOP than GAT.

The mean values of the CCT of the healthy population, in most of the published studies, are between 528μ and 562μ [29,30,31,32], which is similar to those obtained in this study for the healthy control population (556 μ).

### 4.2. Glaucoma, FHG, and Glaucoma Suspect Groups

In the last 15 years, a large number of articles about corneal biomechanical characterization have been published [33,34,35,36,37,38]. In the context of corneal biomechanical parameters of patients with glaucoma, we previously observed a significant decrease in CH when compared to healthy subjects [39,40]. This coincides with the results of other authors [14,41,42,43] who also found a significant decrease in CH in glaucoma, which was particularly evident in cases of congenital glaucoma [22]. The published results of CRF values are not consistent, as some have obtained low values [37] and others have obtained high values [35]. Our study aims to go into greater depth of cases of suspected glaucoma, which are referred to in this study, either owing to a family history of this pathology or to some doubtful sign, such as a suspicious papillary excavation or borderline or high IOP values, but without evident glaucomatous damage that would confirm the diagnosis.

We carried out a statistical study with multiple comparisons in which we obtained results for each group; we compared them with those of the control group, and we also compared them between each other to verify if there were any statistically significant differences. We found no published study that performs a comparative analysis on these groups of patients, so the results may have a special impact on the assessment of this pathology. This study has several limitations, since we did not analyze the evolutionary data of patients with glaucoma, nor did we differentiate pigmentary from pseudoexfoliative glaucoma; we did not include a group of normal-tension glaucoma, nor did we assess cases of angle-closure glaucoma.

#### 4.2.1. CH

In this study, we verified the existence of decreased CH in the DG group compared to the control group; however, we did not find statistically significant differences in CH values in the three groups studied for suspected glaucoma and FHG when compared to the control group. These groups did not present evident glaucomatous lesions in the visual field or in the OCT fiber study. Consequently, CH could be a promising indicator to predict the progression of glaucoma, since it determines a specific biomechanical condition that is found in the cornea, but that could also affect other ocular structures, conditioning a special susceptibility to suffer glaucomatous damage. Recent studies suggest a possible relationship between lower CH values and structural changes in the optic nerve associated with glaucomatous damage. The relationship of low CH values with mean cup depth, linear cup-to-disc ratio, and greater posterior displacement of the lamina cribrosa has been evidenced [44,45,46]. Corneal hysteresis may provide insight into the biomechanical properties of the optic nerve and its supporting structures.

Some research indicates that eyes with higher IOP have lower CH values, and that the therapeutic manipulation of IOP can induce an inverse response in CH. It has been suggested that it may be due to the effect of prostaglandin therapy. Nevertheless, Meda et al. [47] do not support this hypothesis and suggest that this increase in CH may be due to IOP control. Furthermore, other medical and surgical treatments have shown to increase CH. Sun et al. [48] found that CH was significantly lower in eyes with chronic angle-closure glaucoma (CACG) compared to the contralateral eye and the control group of normal eyes. On the other hand, in this prospective study, it was found that trabeculectomy decreased the mean IOP from 31.5 to 11.5 mmHg, and that CH increased from 6.8 to 9.2 mmHg in the same period, although CH remained lower than in the contralateral eye without CACG. This study reflects the apparent dependence of CH according to IOP, and the probability that the regression of high IOP increases the mean of CH in this type of patients.

#### 4.2.2. CRF

In this study, the values of CRF in the DG group showed higher values than the control group, but we found no statistically significant differences (*p* > 0.05). FHG, GLD, and OHT groups showed higher CRF values than the control group, with statistically significant differences. The OHT group showed the highest CRF values. Pillunat et al. [37] found that glaucoma patients had the lowest CRF values (9.07 ± 1.93 mmHg) in comparison to controls (10.2 ± 1.9 mmHg) and OHT patients (CRF: 10.6 ± 2.1 mmHg). However, Kaushik et al. found that CRF was significantly higher in POAG and OHT, which matches our results.

#### 4.2.3. CCT

Regarding CCT, in the DG group we found no differences in the CCT of DG patients compared to those of the control group, which coincides with the results of other authors [14,22,38,42]. Although low CCT influences an underestimation of IOP by GAT [16], our glaucoma patients did not present different CCT values from those of the normal population, and the underestimation of IOP by GAT (IOPcc higher than IOPg) in the DG group could be related to other corneal biomechanical conditions, such as CH.

#### 4.2.4. CH and CCT

Several studies have analyzed the association between CH and the visual field and optic nerve deterioration in glaucomatous patients [46,49,50], but the understanding of the direct importance of CH for glaucoma, and especially its relationship with CCT, is still evolving. Although it is true that low CCT has been related to the risk of OHT progression to glaucoma and to the progression of glaucomatous visual field loss, the results of our study agree with those of other authors, such as Congdon et al. [49], who found an association of low CH values with greater progression of the glaucomatous visual field, regardless of the CCT values, in their retrospective observational clinical study. It should be taken into account that CH is a direct estimate of an aspect of ocular biomechanics measured in the cornea, while CCT represents only one parameter that affects biomechanics. Wells et al. [46] found a relationship between low CH and the existence of greater deformation of the optic nerve after hyperpressure caused by suction in patients with glaucoma, but not with CCT.

The importance of measurable biomechanical parameters of the cornea has not yet been fully clarified. We know that CCT influences the estimation of a real IOP. In fact, there are structurally strong, thick corneas (high CCT, CH, and CRF), as in cases of hyperopic patients or OHT with high pachymetry values [51,52,53], where the IOPcc values are lower than the GAT or IOPg. There are also structurally weak, thick corneas, as in cases of endothelial dystrophy, bullous keratopathy, or rejection in keratoplasties [12,54]. In Fuchs’ endothelial dystrophy, it has been shown that, when a high CCT value is due to corneal edema, there is a decrease in the CH and CRF biomechanical parameters. In these cases, there is no overestimation of IOP due to the increase in CCT, quite the opposite. The GAT in these patients underestimates the IOP and the IOPcc values show a higher real IOP [12].

The ORA can provide additional interesting information in the study of glaucoma patients. On the one hand, it provides an IOPcc value that is less influenced by the biomechanical properties of the cornea, which helps assess each case and, furthermore, it indicates CH values that can give guidance in the control of patients. The existence of a low CH value can be considered a risk factor for glaucoma progression. Likewise, in cases of OHT, the presence of high biomechanical values of CH, CRF, and CCT may indicate a low risk of progression to glaucoma; these cases usually present lower IOPcc values (in the normal range) than those of IOPg and GAT.

The present study provides the following conclusions on corneal biomechanics and IOP with the ORA:

1. A mean CH and CRF value of 10.75 mmHg was established in the healthy control population, which can be a reference for the Spanish population. The mean IOPg (15.63 mmHg) and IOPcc (15.72) values estimated by the ORA, in patients without ocular pathology, were similar to the mean GAT values. IOPg and IOPcc values were similar when the biomechanical properties of the cornea were within normal limits.
2. The IOPg and IOPcc means were significantly higher than those of the control group in all glaucoma and glaucoma suspect groups. There was a significant decrease in CH in the DG group compared to the control group, and with respect to the three glaucoma suspect groups. However, the CH values in the three glaucoma suspect groups (FHG, GLD, and OHT) did not show statistically significant differences between them, or with respect to the control group. No statistically significant differences in CRF values between the DG group and the control group were found. However, elevated CRF values in all the glaucoma suspect groups were found, such differences being statistically significant with respect to the control group. There was no CCT alteration in the DG group. POAG showed its own biomechanical profile (normal or high CCT, normal or high CRF, and low CH with IOPcc > IOPg).
3. The OHT and FHG suspect groups presented higher CRF values than the DG group and the differences were statistically significant. The mean CCT values were higher than the control group in all groups, although there were no statistically significant differences. Ocular hypertension suspect cases showed the highest CCT values. OHT showed its own biomechanical profile (high CCT, high CRF, and normal or high CH with IOPcc < IOPg).

Finally, future studies could be performed to compare the biomechanical characterization of the cornea by two different devices, namely ORA and CORVIS-ST. To date, no study has found a relationship between the parameters they report. The contribution to diagnosis of CORVIS-ST has been studied mainly for corneal ectasia, but there is a lack of data about the differential role of CORVIS parameters in the glaucoma spectrum.

## Figures and Tables

**Table 1:** Demographics of the control and the four diagnosis groups included in the study (DG, FHG, GLD, and OHT). Gender is summarized by frequency and percentage; age by mean ± SD.

	Control (*n* = 574)	DG (*n* = 147)	FHG (*n* = 78)	GLD (*n* = 90)	OHT (*n* = 176)	Total (*n* = 1065)
Female	291 (50.7%)	65 (44.2%)	56 (71.8%)	71 (78.9%)	95 (54%)	578 (54.3%)
Male	283 (49.3%)	82 (55.8%)	22 (28.2%)	19 (21.1%)	81 (46%)	487 (45.7%)
Age (years)	39 ± 15	56 ± 16	47 ± 15	48 ± 14	51 ± 13	46 ± 17

DG—diagnosed glaucoma; FHG—family history of glaucoma; GLD—glaucoma-like optic discs; OHT—ocular hypertension; SD—standard deviation.

**Table 2:** Descriptive statistics of the control and the four diagnosis groups included in the study (DG, FHG, GLD, and OHT). Variables summarized by mean ± SD.

	Control (*n* = 574)	DG (*n* = 147)	FHG (*n* = 78)	GLD (*n* = 90)	OHT (*n* = 176)
IOPg (mmHg)	15.63 ± 3.1	20.07 ± 3.6	21.01 ± 4.0	18.48 ± 3.6	21.76 ± 3.6
IOPcc (mmHg)	15.72 ± 3.0	20.68 ± 3.7	20.41 ± 3.8	18.30 ± 3.9	21.22 ± 3.8
CH (mmHg)	10.75 ± 1.5	9.69 ± 1.9	10.70 ± 1.7	10.63 ± 1.9	10.54 ± 1.8
CRF (mmHg)	10.75 ± 1.6	11.18 ± 2.0	12.32 ± 1.9	11.50 ± 1.9	12.41 ± 1.8
CCT (μ)	556.8 ± 35.3	562.6 ± 39.6	576.3 ± 38.3	569.5 ± 31.5	570.3 ± 34.7

DG—diagnosed glaucoma; FHG—family history of glaucoma; GLD—glaucoma-like optic discs; OHT—ocular hypertension; SD—standard deviation. IOPg: Goldmann-correlated intraocular pressure, IOPcc: corneal-compensated IOP, CH: Corneal Hysteresis, CRF: Corneal Resistance Factor, CCT: Central Corneal Thickness.

**Table 3:** Statistical results using ANOVA multiple comparisons test for the CH variable.

Dependent Variable	(I) Pathology	(J) Pathology	Mean Difference (I–J)	SE	*p* Value	95% CI	
						Upper Limit	Lower Limit
CH	Control	DG	1.053 (*)	0.174	0.000 *	0.502	1.604
		FHG	0.052	0.207	1.000	−0.611	0.717
		GLD	0.119	0.216	1.000	−0.572	0.811
		OHT	0.210	0.153	0.995	−0.272	0.693
	DG	Control	−1.053 (*)	0.174	0.000 *	−1.604	−0.502
		FHG	−1.000 (*)	0.254	0.003 *	−1.807	−0.194
		GLD	−0.934 (*)	0.262	0.013 *	−1.763	−0.104
		OHT	−0.842 (*)	0.213	0.003 *	−1.513	−0.172
	FHG	Control	−0.052	0.207	1.000	−0.717	0.611
		DG	1.000 (*)	0.254	0.003 *	0.194	1.807
		GLD	0.066	0.285	1.000	−0.836	0.970
		OHT	0.157	0.240	1.000	−0.605	0.921
	GLD	Control	−0.119	0.216	1.000	−0.811	0.572
		DG	0.934 (*)	0.262	0.013 *	0.104	1.763
		FHG	−0.066	0.285	1.000	−0.970	0.836
		OHT	0.091	0.248	1.000	−0.696	0.879
	OHT	Control	−0.210	0.153	0.995	−0.693	0.272
		DG	0.842 (*)	0.213	0.003 *	0.172	1.513
		FHG	−0.157	0.240	1.000	−0.921	0.605
		GLD	−0.091	0.248	1.000	−0.879	0.696

SE—standard error, * Statistical significance (*p* < 0.05), CI—confidence interval, DG—diagnosed glaucoma; FHG—family history of glaucoma; GLD—glaucoma-like optic discs; OHT—ocular hypertension.

**Table 4:** Statistical results using ANOVA multiple comparisons test for the CRF variable.

Dependent Variable	(I) Pathology	(J) Pathology	Mean Difference (I–J)	SE	*p*-Value	95% CI	
						Upper Limit	Lower Limit
CRF	Control	DG	−0.429	0.180	0.403	−0.998	0.140
		FHG	−1.562 (*)	0.235	0.000 *	−2.317	−0.808
		GLD	−0.745 (*)	0.214	0.020 *	−1.430	−0.060
		OHT	−1.653 (*)	0.155	0.000 *	−2.144	−1.163
	DG	Control	0.429	0.180	0.403	−0.140	0.998
		FHG	−1.133 (*)	0.279	0.002 *	−2.018	−0.249
		GLD	−0.316	0.262	0.999	−1.145	0.511
		OHT	−1.224 (*)	0.216	0.000 *	−1.906	−0.543
	FHG	Control	1.562 (*)	0.235	0.000 *	0.808	2.317
		DG	1.133 (*)	0.279	0.002 *	0.249	2.018
		GLD	0.816	0.302	0.193	−0.141	1.775
		OHT	−0.091	0.263	1.000	−0.929	0.747
	GLD	Control	0.745 (*)	0.214	0.020 *	0.060	1.430
		DG	0.316	0.262	0.999	−0.511	1.145
		FHG	−0.816	0.302	0.193	−1.775	0.141
		OHT	−0.908 (*)	0.245	0.008 *	−1.686	−0.129
	OHT	Control	1.653 (*)	0.155	0.000 *	1.163	2.144
		DG	1.224 (*)	0.216	0.000 *	0.543	1.906
		FHG	0.091	0.263	1.000	−0.747	0.929
		GLD	0.908 (*)	0.245	0.008 *	0.129	1.686

SE—standard error, * Statistical significance (*p* < 0.05), CI—confidence interval, DG—diagnosed glaucoma; FHG—family history of glaucoma; GLD—glaucoma-like optic discs; OHT—ocular hypertension.

**Table 5:** Statistical results using ANOVA multiple comparisons test for the IOPg variable.

Dependent Variable	(I) Pathology	(J) Pathology	Mean Difference (I–J)	SE	*p*-Value	95% CI	
						Upper Limit	Lower Limit
IOPg	Control	DG	−4.435 (*)	0.328	0.000 *	−5.472	−3.397
		FHG	−5.378 (*)	0.474	0.000 *	−6.902	−3.854
		GLD	−2.844 (*)	0.408	0.000 *	−4.149	−1.539
		OHT	−6.130 (*)	0.302	0.000 *	−7.083	−5.177
	DG	Control	4.435 (*)	0.328	0.000 *	3.397	5.472
		FHG	−0.943	0.546	0.921	−2.680	0.792
		GLD	1.590 (*)	0.490	0.039 *	0.038	3.142
		OHT	−1.695 (*)	0.406	0.001 *	−2.974	−0.415
	FHG	Control	5.378 (*)	0.474	0.000 *	3.854	6.902
		DG	0.943	0.546	0.921	−0.792	2.680
		GLD	2.534 (*)	0.598	0.001 *	0.636	4.432
		OHT	−0.751	0.531	0.992	−2.442	0.939
	GLD	Control	2.844 (*)	0.408	0.000 *	1.539	4.149
		DG	−1.590 (*)	0.490	0.039 *	−3.142	−0.038
		FHG	−2.534 (*)	0.598	0.001 *	−4.432	−0.636
		OHT	−3.285 (*)	0.474	0.000 *	−4.785	−1.785
	OHT	Control	6.130 (*)	0.302	0.000 *	5.177	7.083
		DG	1.695 (*)	0.406	0.001 *	0.415	2.974
		FHG	0.751	0.531	0.992	−0.939	2.442
		GLD	3.285 (*)	0.474	0.000 *	1.785	4.785

SE—standard error. * Statistical significance (*p* < 0.05). CI—confidence interval; DG—diagnosed glaucoma; FHG—family history of glaucoma; GLD—glaucoma-like optic discs; OHT—ocular hypertension.

**Table 6:** Statistical results using ANOVA multiple comparisons test for the IOPcc variable.

Dependent Variable	(I) Pathology	(J) Pathology	Mean Difference (I–J)	SE	*p*-Value	95% CI	
						Upper Limit	Lower Limit
IOPcc	Control	DG	−4.954 (*)	0.335	0.000 *	−6.014	−3.895
		FHG	−4.686 (*)	0.450	0.000 *	−6.131	−3.241
		GLD	−2.577 (*)	0.434	0.000 *	−3.966	−1.187
		OHT	−5.503 (*)	0.314	0.000 *	−6.494	−4.513
	DG	Control	4.954 (*)	0.335	0.000 *	3.895	6.014
		FHG	0.268	0.531	1.000	−1.416	1.953
		GLD	2.377 (*)	0.518	0.000 *	0.737	4.017
		OHT	−0.548	0.422	0.998	−1.877	0.779
	FHG	Control	4.686 (*)	0.450	0.000 *	3.241	6.131
		DG	−0.268	0.531	1.000	−1.953	1.416
		GLD	2.109 (*)	0.599	0.015 *	0.211	4.007
		OHT	−0.817	0.518	0.969	−2.462	0.828
	GLD	Control	2.577 (*)	0.434	0.000 *	1.187	3.966
		DG	−2.377 (*)	0.518	0.000 *	−4.017	−0.737
		FHG	−2.109 (*)	0.599	0.015 *	−4.007	−0.211
		OHT	−2.926 (*)	0.505	0.000 *	−4.525	−1.327
	OHT	Control	5.503 (*)	0.314	0.000 *	4.513	6.494
		DG	0.548	0.422	0.998	−0.779	1.877
		FHG	0.817	0.518	0.969	−0.828	2.462
		GLD	2.926 (*)	0.505	0.000 *	1.327	4.525

SE—standard error. * Statistical significance (*p* < 0.05). CI—confidence interval. DG—diagnosed glaucoma; FHG—family history of glaucoma; GLD—glaucoma-like optic discs; OHT—ocular hypertension.

**Table 7:** Statistical results using ANOVA multiple comparisons test for the CCT variable.

Dependent Variable	(I) Pathology	(J) Pathology	Mean Difference (I–J)	SE	*p*-Value	95% CI	
						Upper Limit	Lower Limit
CCT	Control	DG	−5.80	4.36	0.997	−19.71	8.10
		FHG	−19.45 *	5.01	0.006 *	−35.64	−3.27
		GLD	−12.64	4.34	0.122	−26.63	1.35
		OHT	−13.47 *	3.52	0.005 *	−24.62	−2.33
	DG	Control	5.80	4.36	0.997	−8.10	19.71
		FHG	−13.64	6.26	0.585	−33.54	6.24
		GLD	−6.83	5.73	0.999	−25.02	11.35
		OHT	−7.67	5.14	0.984	−23.92	8.58
	FHG	Control	19.45 *	5.01	0.006 *	3.27	35.64
		DG	13.64	6.26	0.585	−6.24	33.54
		GLD	6.81	6.24	1.000	−13.07	26.70
		OHT	5.97	5.70	1.000	−12.21	24.16
	GLD	Control	12.64	4.34	0.122	−1.35	26.63
		DG	6.83	5.73	0.999	−11.35	25.02
		FHG	−6.81	6.24	1.000	−26.70	13.07
		OHT	−0.83	5.11	1.000	−17.12	15.44
	OHT	Control	13.47 *	3.52	0.005 *	2.33	24.62
		DG	7.67	5.14	0.984	−8.58	23.92
		FHG	−5.97	5.70	1.000	−24.16	12.21
		GLD	0.83	5.11	1.000	−15.44	17.12

SE—standard error. * Statistical significance (*p* < 0.05). CI—confidence interval. DG—diagnosed glaucoma; FHG—family history of glaucoma; GLD—glaucoma-like optic discs; OHT—ocular hypertension.

## Data Availability

Data available on request due to restrictions of privacy.
